# Awards for Valuable Services in Connection with the War

**Published:** 1917-03-10

**Authors:** 


### ROYAL RED CROSS (Second Class).

### Awards for Valuable Services in Connection with the War.

(*Continued from March 3, page vi.*)

C.

C. Cameron, Nursing Sister, Canadian Nursing Service; J. C. Cameron, Staff Nurse, Q.A.I.M.N.S.R., Wharncliffe War Hosp., Middlewood Road, Sheffield; F. Campbell, Staff Nurse, Q.A.I.M.N.S.R., Pavilion and York Place Hosp., Brighton; B. Carley, Sister, T.F.N.S., 1st Eastern Gen. Hosp., Cambridge; C. Carmichael, Staff Nurse, The Horton (County of London) War Hospital, Epsom; I. Carnaghan, Sister, Q.A.I.M.N.S.R., Kinmell Park Camp Mil. Hosp., Abergele; I. Carruthers, Staff Nurse (Acting Matron), Q.A.I.M.N.S., Mil. Hosp., Caterham; and C. Carvel, Staff Nurse, Edinburgh War Hosp., Bangour; Mrs. G. Carswell, Sister, Edinburgh War Hosp., Bangour; G. Carter, Sister, Egginton Hall Hospital, Derby, and St. Thomas's Hosp, London; H. C. Chalmers, Staff Nurse, T.F.N.S., 2nd Scottish General Hosp., Craigleith, Edinburgh; G. W. Chamberlain, Acting Sister, Q.A.I.M.N.S., Queen Alexandra Mil. Hosp., Grosvenor Road, London; W. Chapman, Sister, Q.A.I.M.N.S.R., Mil. Hosp., Grantham; M. K. Chatterley, Asst. Matron, T.F.N.S., 1st Southern Gen. Hosp., Edgbaston, Birmingham; Elizabeth Charles, Staff Nurse, Norfolk War Hosp., Thorpe, Norwich; S. Cherry, Sister, 1st Western Gen. Hospital, Fazakerley, Liverpool; A. Clark, Sister, Q.A.I.M.N.S.R., Barnet War Hosp.; E. Clarke, Sister, Q.A.I.M.N.S.R., King George V. Hospital, Dublin; M. Clarke, Staff Nurse, Q.A.I.M.N.S.R., King George Hospital, Stamford Street, London; M. C. Clarke, Sister, Q.A.I.M.N.S.R., Military Hospital, Colchester; M. Clint, Nursing Sister, Can. Nursing Service; L. Clough, Sister, 2nd Western Gen. Hosp., Manchester; M. M. Cocking, Staff Nurse, Q.A.I.M.N.S.R., Mil. Isolation Hospital, Aldershot; A. M. Collins, Staff Nurse, Q.A.I.M.N.S.R., Mil. Hosp., Frensham Hill, Farnham; M. Cook, Staff Nurse, Huddersfield War Hosp.; L. M. Cook, Sister, East Suffolk and Ipswich Hosp., Ipswich; S. Cooke, Staff Nurse, 2nd Western Gen. Hosp., Manchester; H. O. Cooper, Sister, The Horton (County of London) War Hosp., Epsom; M. E. Cooper, Asst. Matron, Q.A.I.M.N.S.R., Mil. Hosp., Colchester; A. M. Copper, Matron, Aust. Army Nursing Service; E. Cornwell, Matron, Aust. Army Nursing Service; A. Cowie, Sister, Leith War Hosp., Leith; C. C. Cox, Staff Nurse (Acting Sister), Q.A.I.M.N.S.R., Mil. Hosp., Fargo, Wilts; C. Craig, Matron, T.F.N.S., 1st Scottish Gen. Hosp., Aberdeen; M. L. Craven, Staff Nurse, Aust. N.S., Lord Derby War Hosp., Warrington; E. Creech, Sister, Q.A.I.M.N.S.R., The King George Hosp., Stamford Street, London; M. H. Crook, Asst. Matron, 1st Western Gen. Hosp., Fazakerley, Liverpool; M. R. O'H. Cussen, Sister, Mercy Hosp., Cork.

D.

M. M. Dalrymple, Staff Nurse (Acting Sister), Q.A.I.M.N.S.R., Mil. Hosp., Fargo, Wilts; M. Dando, Sister, 1st Western Gen. Hosp., Fazakerley, Liverpool; K. M. Daniel, Sister 3rd Western Gen. Hosp., Cardiff; E. A. Davies, Sister, Gilroes Hosp., Leicester; J. Davies (now Mrs. Nicholas), Acting Sister, Q.A.I.M.N.S.R., Alexandra Mil. Hosp., Cosham; I. E. Dawson, Sister, Q.A.I.M.N.S.R., Royal Victoria Hosp., Netley; and R. Day, Sister, Gen. Hosp., Birmingham; Mrs. L. Dent, Commandant V.A.D., City of London 10; E. Devlin, Sister, T.F.N.S., 1st Southern Gen. Hosp., Edgbaston, Birmingham; C. C. Douet, Sister, Q.A.I.M.N.S.R., Park Hall Camp Mil. Hosp., Oswestry; M. Dow, Acting Matron, Q.A.I.M.N.S.R., Mil. Hosp., Aylesbury; E. Drysdale, Nursing Sister, Can. Nursing Service; J. Dunlop, Sister, T.F.N.S., 4th Scottish Gen. Hosp., Stobhill, Glasgow; and B. M. Duff, Sister, Q.A.I.M.N.S.R., King George V. Hosp., Dublin; Mrs. M. I. Duffus, Sister, Red Cross Hosp., Netley; A. Dunbabin, Sister, Nell Lane Auxiliary Mil. Hosp., West Didsbury, Manchester; and G. Dunsford, Sister, T.F.N.S., 2nd London Gen. Hosp., St. Mark's College, Chelsea, London.

E.

E. Ealand, Staff Nurse, T.F.N.S., 1st Eastern General Hospital, Cambridge; and G. I. Echlin, Sister, Aust. Army Nursing Service, Royal Victoria Hosp., Netley; Mrs. J. B. Edgar, Asst. Matron, T.F.N.S., 4th Scottish General Hospital, Stobhill, Glasgow; D. Edgley, Sister, T.F.N.S., 4th Southern Gen. Hosp., Plymouth; A. Edwards, Sister, Salford Royal Hosp., Manchester; M. F. Eldridge, Staff Nurse, Aust. Army Nursing Service; R. Elliott, Sister, Worcester Gen. Hosp., Worcester; A. Ellis, Staff Nurse, Q.A.I.M.N.S.R., Malta; E. C. Ellis, Sister (Acting Matron), Q.A.I.M.N.S.R.; M. P. Ellis, Nursing Sister, Can. Nursing Serv.; K. Elphick, Matron, The Lady Forester Convalescent Home, Llandudno; M. Elsdon, Sister, The Horton (County of London) War Hosp., Epsom; M. C. English, Nursing Sister, Can. Nursing Service; L. A. Ephgrave, Staff Nurse (Acting Matron), Q.A.I.M.N.S., Retd., Malta; J. I. Ettles, Acting Matron, Q.A.I.M.N.S.R., Mil. Hosp., Sheerness; B. Evans, Sister, T.F.N.S., 1st Southern Gen. Hosp., Edgbaston, Birmingham; E. G. Evans, Matron, Welsh Red Cross Hospital, Netley; and J. A. Evans, Sister, Q.A.I.M.N.S., Croydon War Hosp.

F.

K. J. Fancourt, Sister, Q.A.I.M.N.S.R., Wharncliffe War Hospital, Middlewood Road, Sheffield; and A. Farren, Sister, T.F.N.S., 2nd Eastern Gen. Hosp., Brighton; Mrs. H. Ferguson, Matron, Gilford Branch Hospital, Belfast District; M. S. Finlay, House Matron, County of Middlesex War Hospital, Napsbury, St. Albans; Mrs. M. C. A. Fishbourne, Asst. Matron, Q.A.I.M.N.S.R., Pavilion and York Place Hospital, Brighton; M. Fitz-Henry, 1st Assistant Matron, The Horton (County of London) War Hosp., Epsom; E. G. Fleming, Sister, Aust. Army Nursing Service; M. A. Fogarty, Sister, South Inf., Cork District; A. Foley, Sister, 3rd Western Gen. Hosp., Cardiff; H. L. Fowlds, Nursing Sister, Can. Nursing Service; S. H. Foxe, Sister, The Horton (County of London) War Hosp., Epsom; S. A. Francis, Ward Sister, Royal Infirmary, Leicester; A. W. Fraser, Acting Matron, Q.A.I.M.N.S.R., Mil. Hosp., Cromarty; E. Fraser, Sister, Scottish National Red Cross Hosp., Bellahouston, Glasgow; and W. Furze, Sister, University College Hosp., Gower Street, London.

G.

A. M. Gallop, Nursing Sister, Canadian Nursing Service; E. Gardner, Sister, Brook War Hospital, Woolwich; P. Gibbins, Sister, Q.A.I.M.N.S.R., Mil. Hosp., Ripon; M. McI. P. Gibb, Matron, Smithson War Hosp., Greenock, Renfrewshire; B. L. Gibbon, Sister, Aust. Army Nursing Service, Royal Victoria Hosp., Netley; J. Gibson, Asst. Matron, Scottish National Red Cross Hosp., Bellahouston, Glasgow; F. E. Gill, Sister, T.F.N.S., 5th Southern Gen. Hosp., Fawcett Road, Southsea; P. M. Gill, Sister, Q.A.I.M.N.S.R., The Lord Derby War Hosp., Warrington; N. C. Gillam, Matron, Q.A.I.M.N.S.R., Kinmel Park Camp Mil. Hosp., Abergele; J. M. R. Gilmer, Sister, New Zealand Army Nursing Service; F. M. Gittins, Sister, Q.A.I.M.N.S.R., The Lord Derby War Hosp., Warrington; A. A. Glover, Acting Sister, Q.A.I.M.N.S.R., Alexandra Mil. Hosp., Cosham; H. M. Goldthorpe; Sister, Bethnal Green Mil. Hosp., Cambridge Heath, E.; M. C. Goodhue, Secretary, County of London Branch, British Red Cross Society, and Matron-in-charge City of London Red Cross Hosp., Finsbury Square, N.; L. Gordon, Sister, T.F.N.S., 1st Scottish Hosp., Aberdeen; A. Grattan, Sister, Norfolk and Norwich Hosp., Norwich; G. A. Gray, Nursing Sister, Can. Nursing Service; N. Gray, Sister, Edinburgh War Hosp., Bangour; G. E. Green, Sister, Q.A.I.M.N.S.R., Queen Mary's Mil. Hosp., Whalley, Lancashire; J. Greig, Asst. Matron, City of London Mil. Hosp., Clapton, N.E.; D. Groom, Staff Nurse, Q.A.I.M.N.S., Dartford War Hosp.; and J. E. Guffog, Staff Nurse, T.F.N.S., 2nd Western Gen. Hosp., Manchester.

(*Continued on page vi.*)

